# P-2071. 2023-2024 COVID-19 Vaccine Uptake, Knowledge, and Attitudes among Individuals enrolled in a Household Transmission Study, Tennessee, New York, and Washington, January-April 2024

**DOI:** 10.1093/ofid/ofae631.2227

**Published:** 2025-01-29

**Authors:** J Bradford Bertumen, Sara Benist, Erin South, E Ivy Oyegun, Sarah E Smith-Jeffcoat, Carlos G Grijalva, Yuwei Zhu, Theresa A Scott, Jonathan Schmitz, Helen Y Chu, Ana A Weil, Janet A Englund, Melissa P MacMillan, Melissa Stockwell, Son H McLaren, Ellen Sano, Celibell Vargas, Keipp Talbot, Hannah L Kirking

**Affiliations:** Centers for Disease Control and Prevention, DECATUR, Georgia; Centers For Disease Control and Prevention, Atlanta, Georgia; Centers for Disease Control and Prevention, DECATUR, Georgia; Centers for Disease Control and Prevention, DECATUR, Georgia; Centers for Disease Control and Prevention, DECATUR, Georgia; Vanderbilt University Medical Center, Nashville, Tennessee; Vanderbilt University, Nashville, Tennessee; Vanderbilt University Medical Center, Nashville, Tennessee; Vanderbilt University Medical Center, Nashville, Tennessee; University of Washington, Seattle, WA; University of Washington, Seattle, WA; Seattle Children’s Hospital, Seattle, Washington; University of Washington, Seattle, WA; Columbia University Irving Medical Center, New York City, New York; Columbia University Irving Medical Center, New York City, New York; Columbia University Irving Medical Center, New York City, New York; Columbia University Irving Medical Center, New York City, New York; Vanderbilt University Medical Center, Nashville, Tennessee; Coronavirus and Other Respiratory Viruses Division, National Center for Immunization and Respiratory Diseases, CDC, Atlanta, GA

## Abstract

**Background:**

The updated 2023-2024 Coronavirus Disease 2019 (COVID-19) vaccine became available on September 11, 2023 for persons ≥ 6 months old. Understanding the targeted populations’ knowledge, attitudes, and practices regarding this latest vaccine may identify areas to improve future vaccine uptake.

**Table 1:**
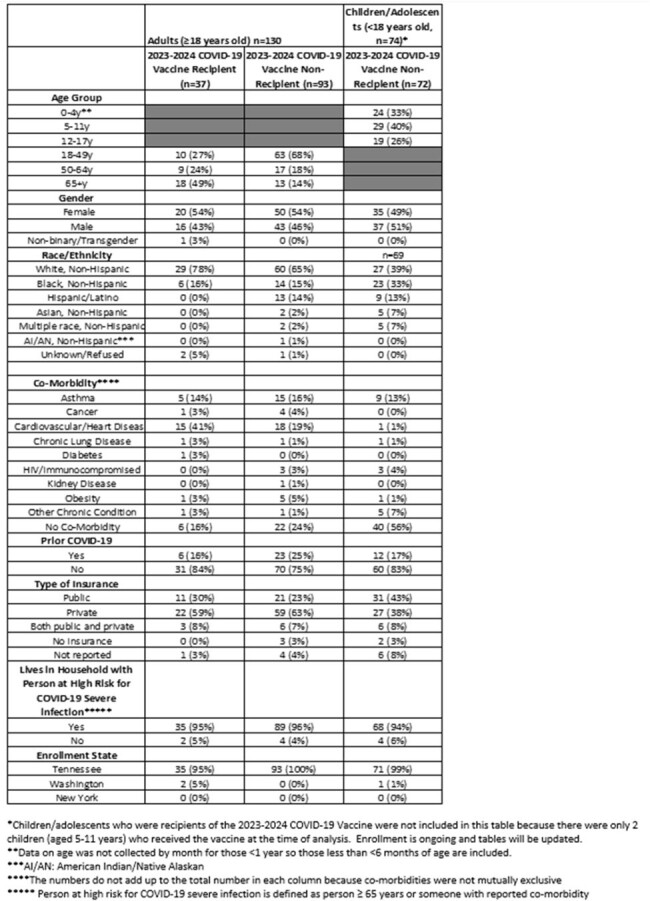
Participants Characteristics Stratified by Age and Receipt of the 2023-2024 COVID-19 Vaccine

**Methods:**

Persons enrolled in a household transmission investigation of respiratory viruses in Tennessee, New York, and Washington from January–April 2024 were asked to complete an enrollment survey, which included patient demographics and clinical history, and knowledge, attitude, and practice (KAP) questions about recent COVID-19 vaccine recommendations. Surveys for children < 13 years old were completed by their parent or guardian. All persons who answered at least one KAP question and who had verified COVID-19 vaccination information were included in this analysis (Figure 1). Characteristics and responses of recipients and non-recipients of the updated COVID-19 vaccine were described.

**Table 2:**
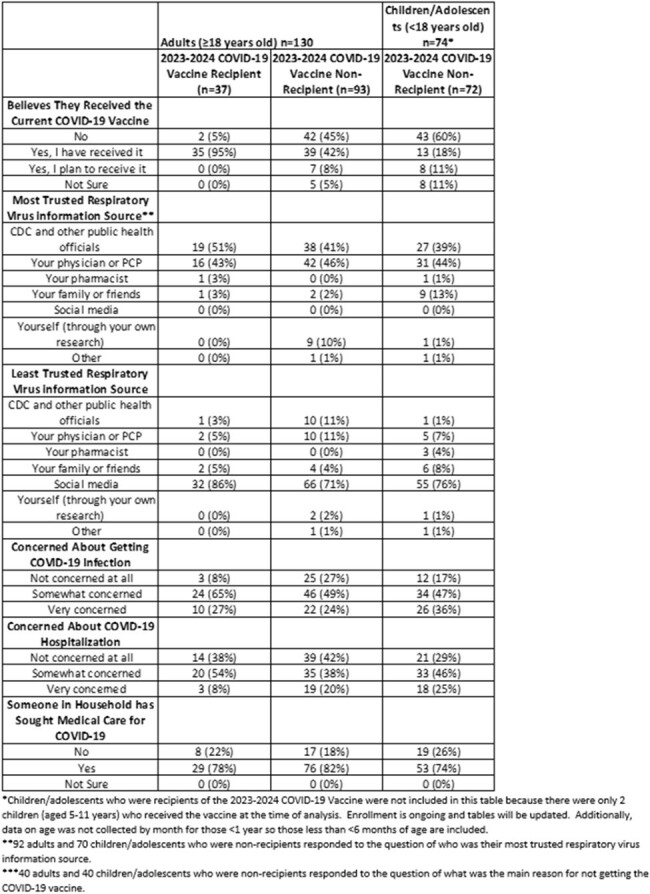
Knowledge, Attitudes and Practices Stratified by Age and Receipt of the 2023-2024 COVID-19 Vaccine

**Results:**

Of 204 individuals included in the analysis, 130 (64%) were adults (≥18 years) and 74 (36%) were children/adolescents (< 18 years) (Figure 1). Ninety-three (72%) adults and 72 (97%) children/adolescents (including everyone aged ≤4 years and 12-17 years) did not receive the 2023-2024 COVID-19 vaccine. Of 93 adults who were non-recipients, those aged 18-49 years (n=63, 68%) represented the most common age group, and 39 (42%) believed they had received the updated vaccine (Table 1 and Table 2). Of the 40 adult non-recipients who responded to the question of why they did not get vaccinated, 13 (33%) stated COVID-19 was not a problem in their community and 8 (20%) adults did not believe the vaccine would prevent COVID-19 or its complications. Of the 40 children/adolescent non-recipients who responded to the same question, 14 (35%) had safety concerns listed as the reason for not receiving the vaccine (Table 2).

**Table 2:**
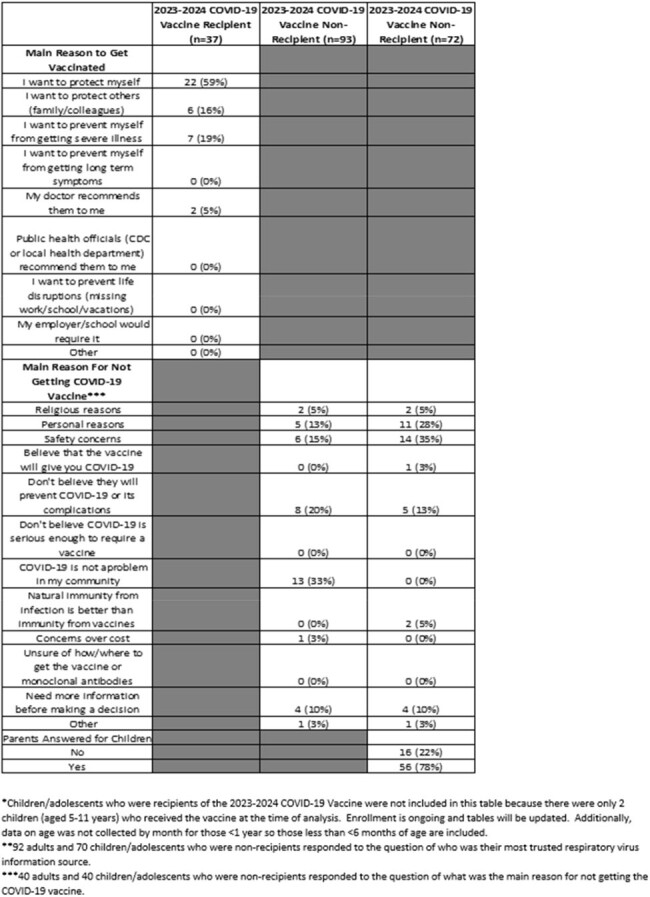
Knowledge, Attitudes and Practices Stratified by Age and Receipt of the 2023-2024 COVID-19 Vaccine (continued)

**Conclusion:**

Uptake of the 2023-2024 COVID-19 vaccine was particularly low in persons aged < 18 years and 18-49 years. Education on the updated vaccination recommendations, the safety of the vaccine, and its effectiveness in preventing complications may improve uptake among these groups. More vaccine uptake and KAP data will be collected with further enrollment.

**Figure 1:**
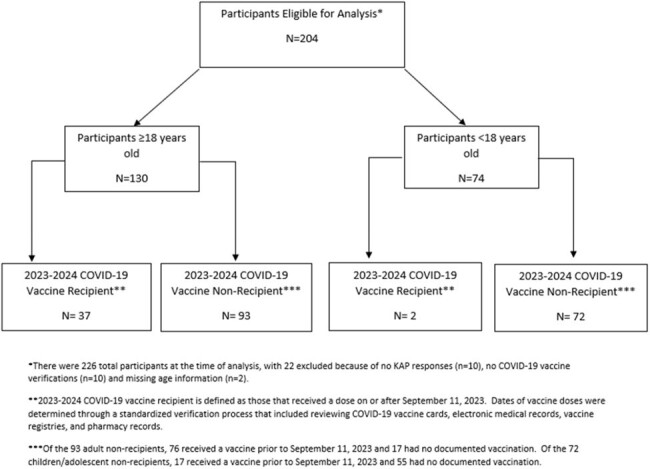
Participant 2023-2024 COVID-19 Vaccine Eligibility and Receipt

**Disclosures:**

Carlos G. Grijalva, MD, MPH, AHRQ: Grant/Research Support|CDC: Grant/Research Support|FDA: Grant/Research Support|Merck: Advisor/Consultant|NIH: Grant/Research Support|SyneosHealth: Grant/Research Support Jonathan Schmitz, MD, PhD, D(ABMM), Biofire/Biomerieux: Grant/Research Support|Genmark/Roche: Grant/Research Support|Pfizer: Honoraria|Vela: Grant/Research Support Helen Y. Chu, MD, MPH, Abbvie: Advisor/Consultant|Merck: Advisor/Consultant|Vir: Advisor/Consultant Janet A. Englund, MD, Abbvie: Advisor/Consultant|AstraZeneca: Advisor/Consultant|AstraZeneca: Grant/Research Support|GlaxoSmithKline: Advisor/Consultant|GlaxoSmithKline: Grant/Research Support|Meissa Vaccines: Advisor/Consultant|Merck: Advisor/Consultant|Pfizer: Board Member|Pfizer: Grant/Research Support|Pfizer: Speaker at meeting|SanofiPasteur: Advisor/Consultant|Shinogi: Advisor/Consultant

